# Impact of Split Dosing the First Rituximab Infusion in Patients with High Lymphocyte Count

**DOI:** 10.3390/curroncol28050349

**Published:** 2021-10-13

**Authors:** Maude Plante, Laurence Garneau, Magali Laprise-Lachance, Pierre Lemieux, Michel Dorval

**Affiliations:** 1 Faculté de Pharmacie, Université Laval, Québec, QC G1V 0A6, Canada; laurence.garneau@ssss.gouv.qc.ca (L.G.); Michel.Dorval@crchudequebec.ulaval.ca (M.D.); 2 Département de Pharmacie, Institut Universitaire en Santé Mentale de Québec, Centre Intégré Universitaire de Santé et de Services Sociaux de la Capitale-Nationale, Québec, QC G1J 2G3, Canada; 3 Département de Pharmacie, Hôpital Sainte-Croix, Centre Intégré Universitaire de Santé et de Services Sociaux de la Mauricie-et-du-Centre-du-Québec, Drummondville, QC J2B 1C1, Canada; 4 Département de Pharmacie, Hôtel-Dieu de Lévis, Centre Intégré de Santé et de Services Sociaux de Chaudière-Appalaches, Lévis, QC G6V 3Z1, Canada; Magali_Laprise-Lachance@ssss.gouv.qc.ca; 5 Département de Pharmacie, Centre Hospitalier Affilié Universitaire Régional de Trois-Rivières, Centre Intégré Universitaire de Santé et de Services Sociaux de la Mauricie-et-du-Centre-du-Québec, Trois-Rivières, QC G8Z 3R9, Canada; Pierre.Lemieux@ssss.gouv.qc.ca; 6 Axe Oncologie, Centre de Recherche du CHU du Québec-Université Laval, Québec, QC G1S 4L8, Canada; 7 Centre de Recherche du Centre Intégré de Santé et de Services Sociaux de Chaudière-Appalaches, Lévis, QC G6V 3Z1, Canada

**Keywords:** fractionated dosing schedule, high lymphocyte count, infusion-related reactions, rituximab, split dosing

## Abstract

The most common adverse reactions to rituximab are infusion-related reactions (IRR). We evaluated the efficacy of split dosing the first rituximab infusion over two days to reduce IRR incidence in patients with hematological cancer and a high lymphocyte count. This is a retrospective observational study conducted in two healthcare centers in Quebec, Canada. The study enrolled patients with white blood cell counts ≥25.0 × 10^9^/L who received their first rituximab dose for hematological cancer between December 2007 and May 2020. One healthcare center used asymmetrical split dosing, while the other used symmetrical split dosing. A total of 183 treatment episodes were collected from 143 patients. Among patients who received a fractionated dosing schedule, 42% developed an IRR from the first rituximab infusion compared with 50% for the standard protocol (adjusted relative risk, 0.89; *p* = 0.540). No significant difference was observed in IRR severity between either groups. However, 24% of patients who received the asymmetrical protocol developed an IRR compared to 68% for the symmetrical protocol (adjusted relative risk, 0.32; *p* = 0.003). These results suggest that an asymmetrical split dosing could be effective in reducing the incidence of IRR and is preferable to a symmetrical one.

## 1. Introduction

Rituximab is a chimeric monoclonal antibody directed against the CD20 antigen of normal and neoplastic B cells [1]. The binding of rituximab to CD20 is thought to cause tumor lysis by activating the complement cascade (complement-mediated cytotoxicity) and immune effector cells (antibody-dependent cell-mediated cytotoxicity) [2]. Initially approved in the United States, in 1997, to treat relapsed indolent non-Hodgkin lymphoma (NHL), rituximab is officially approved in Canada to treat chronic lymphocytic leukemia (CLL) as well as several types of NHL [1,3]. 

The most common adverse reactions with this molecule are infusion-related reactions (IRR) [4]. They can occur in up to 77% of patients with hematologic malignancies during the first infusion, usually within the first two hours. The risk decreases with each subsequent administration to less than 14% at the eighth infusion [1,5,6]. Mild to moderate IRR can include fever, chills, pruritus, skin rash, nausea, headaches, etc. Severe reactions can involve hypotension, bronchospasm, hypoxia, and angioedema [1,7]. Fatal IRR occurs in less than 0.07% of cases [1]. 

IRR are autoimmune manifestations that can be caused by several mechanisms that are complex to differentiate given their similar clinical presentation [8]. The most likely mechanism appears to be cytokine release syndrome [8,9]. An effector immune cell call occurs upon the binding of a monoclonal antibody to its target cell, in this case, the CD20 antigen on the surface of B cells. The effector immune cell can then recognize the target cell and destroy it by cytolysis or phagocytosis. This destruction leads to the release of cytokine into the circulation, which is greatest during the very first infusion of a monoclonal antibody given the greater tumor load and presence of target cells, but then decreases during subsequent infusions [9].

While cytokine release syndrome is the most accepted mechanism for explaining rituximab-induced IRR [8,10], risk factors for developing IRR are not fully understood. However, the most frequently reported risk factor is a high lymphocyte count, which indicates a high number of circulating malignant cells [1,9,11,12]. Patients with a lymphocyte count exceeding 50.0 × 10^9^/L, or even 25.0 × 10^9^/L, seem to be at greater risk [1,9,11,12]. 

Several strategies are recommended to prevent IRR, such as the use of premedication consisting of an antihistamine, an antipyretic and a glucocorticoid, as well as gradually increasing the infusion rate [1]. A split dosing over two days during the first rituximab cycle is another strategy that can be used in patients with high lymphocyte count [1,11,12]. This approach is used in two hospitals in the province of Quebec, the Hôtel-Dieu de Lévis (HDL) and the Centre hospitalier affilié universitaire régional (CHAUR) in Trois-Rivières. Since 2013, these two centers have gradually adopted a two-day fractionated dosing schedule for patients with a white blood cell (WBC) count ≥25.0 × 10^9^/L. Dosage regimens differ significantly between these two centers, as no specific protocol is currently recommended in the literature. 

To our knowledge, no studies have yet assessed the efficacy of a fractionated dosing schedule compared to standard dosing, although, this strategy is often recommended and already employed in multiple hospitals [1,11,12]. This study aimed to assess the impact of a split-dosing schedule on the risk and severity of IRR during the first dose of rituximab compared to the standard infusion schedule in patients with a high lymphocyte count. We also compared IRR incidence between the fractionated dosing schedule groups in each center. 

## 2. Materials and Methods

### 2.1. Study Design

This retrospective observational cohort study was conducted in HDL and CHAUR, Quebec, Canada. Patients were divided into four treatment groups, according to infusion protocol and treatment center: HDL fractionated dosing schedule, HDL standard protocol, CHAUR fractionated dosing schedule, and CHAUR standard protocol. The standard protocol in both centers was the one recommended in the rituximab monograph [1] and consisted of a dose of 375 mg/m^2^ given on day 1 at an initial infusion rate of 50 mg/h, which could be increased in 50 mg/h increments every 30 min, in the absence of IRR, to a maximum of 400 mg/h. CHAUR used a symmetrical split dosing of 187.5 mg/m^2^ on days 1 and 2 of the first cycle, with the same infusion rate as the standard protocol. On the other hand, HDL used an asymmetrical split dosing of 50 mg/m^2^ on day 1 with an initial infusion rate of 10 mg/h, which could be increased in 10 mg/h increments every 30 min to a maximum of 50 mg/h. The remaining 325 mg/m^2^ was given on day 2 with the same infusion rate as the standard protocol. Premedication consisting of an antihistamine, an antipyretic and a glucocorticoid was given to all patients before each rituximab infusion.

### 2.2. Population and Data Collection

The study enrolled consecutive adult patients with high lymphocyte counts (WBC ≥ 25.0 × 10^9^/L) who received a first rituximab cycle for hematological cancer in CHAUR or HDL between 28 December 2007, and 28 May 2020. The WBC count was collected rather than the lymphocyte count, as this is the marker locally used to assess high tumor burden in both study centers. These two markers are analogous and intrinsically related as an increase in the WBC count in monoclonal B cell malignancies (e.g., CLL and NHL) is associated with high lymphocyte predominance [13].

Considering the chronic, and often incurable nature, of CLL and indolent NHL, data regarding several first cycles of rituximab (referred to as “treatment episodes” in this article) for the same patient were collected if treatments were separated by at least six months. Data regarding rituximab maintenance doses were collected if administered more than six months following the last dose. Treatment episodes were not collected if the patient was pregnant at the time of rituximab infusion or if part of the fractionated dosing schedule was given outside the study period. 

Treatment episodes were identified from the pharmacy software used in HDL and CHAUR. Data were collected retrospectively using electronic patient records for all treatment episodes that met the inclusion criteria

### 2.3. Study Assessments

The primary study outcome is IRR incidence. Any documented signs or symptoms occurring during the first rituximab infusion were considered an IRR, except for isolated hypertension that did not require any medical attention. Signs and symptoms were systematically documented by nurses in the electronic patient records. Pharmacist and physician medical notes also provided information regarding reactions. 

Hypotension was defined as a systolic blood pressure ≤ 100 mmHg. Hypertension was defined as an increase in systolic blood pressure ≥25 mmHg or a systolic blood pressure ≥180 mmHg. When a patient developed new signs or symptoms following a first IRR, whether on resuming rituximab infusion or on day 2 of the split dosing protocol, this reaction was considered a recurrence and not a second IRR. 

Finally, to assess IRR severity, we used the IRR grading scale provided by the National Cancer Institute Common Terminology Criteria for Adverse Events (NCI-CTCAE) v5.0, the latest version available at the time the protocol was written [14]. Grade 1 and 2 IRR, according to the NCI-CTCAE scale, were classified as mild to moderate reactions, while grades 3 through 5 were classified as severe reactions. In the case of any doubt about the severity grade, investigators consulted each other until consensus was reached.

### 2.4. Statistical Analysis 

We compared IRR incidence and severity between the fractionated dosing and standard protocol groups of both centers to assess the efficacy of a split dosing schedule. We also compared IRR incidence between the HDL and CHAUR fractionated dosing groups. Because there are two levels of analysis in this study, i.e., the patient and the healthcare center, we calculated the intraclass correlation coefficient for each outcome to verify whether the healthcare center could explain part of the results and, if necessary, to perform multilevel analyses. Since intraclass correlation coefficient values were negligible, standard statistical models were used. 

Logistic regressions were used to estimate a propensity score for each outcome to account for unbalanced variables at the baseline. This propensity score allowed to gather all unbalanced variables at the baseline into one independent variable, thus, reducing the number of variables to be considered in the regression model. The propensity score analysis was performed using SAS software. For each outcome, we used logistic regressions to evaluate the effect of infusion protocols. Each model was adjusted with the corresponding propensity score in multivariate analysis.

Sensitivity analyses were conducted to determine if prior rituximab exposure and a different WBC threshold (≥50.0 × 10^9^/L) would lead to different results. A sensitivity analysis was also conducted, excluding patients with comorbid autoimmune hemolytic anemia. As an exploratory analysis, logistic regressions were used to identify potential risk factors for developing an IRR in our study. 

All statistical analyses were carried out using SPSS and SAS statistical software. Relative risks with 95% confidence intervals were calculated for each outcome with a bilateral significance level of 0.05. Considering our small sample, a significance level of 0.10 was used for bivariate analyses comparing patient characteristics in all four groups.

## 3. Results

### 3.1. Patient Characteristics 

A total of 183 treatment episodes were collected from 143 patients. Patient characteristics are summarized in Table 1. The median age at the first rituximab infusion was 67 years and 30% were women. Approximately 83% of patients had CLL, while the other most common diagnoses were marginal zone lymphoma (6%), follicular lymphoma (4%), and mantle cell lymphoma (4%). Fludarabine–cyclophosphamide–rituximab (FCR) was the most common chemotherapy protocol among all groups combined (*n* = 55). 

Patient characteristics were well-balanced between the standard protocol groups and the fractionated dosing groups, except for chemotherapy protocols (*p* < 0.0001), concomitant lung condition (*p* = 0.0474), and patients’ sex (*p* = 0.0676). Mean WBC count was also significantly higher in the fractionated dosing schedule groups (114.6 × 10^9^/L) compared to the standard protocol (72.4 × 10^9^/L) (*p* = 0.0070). The mean dexamethasone equivalent dose as premedication was identical in both groups (10 mg). 

The hemoglobin median value was statistically different between the fractionated dosing groups of both centers (*p* = 0.0062). The mean dexamethasone equivalent dose was significantly higher in HDL compared to CHAUR (*p* = 0.0002). The mean WBC count was significantly lower in the HDL than the CHAUR fractionated dosing group (*p* = 0.0003). 

A single episode was excluded from this study because it could not be determined whether the patient’s symptoms were caused by an IRR or pneumonia detected early after rituximab infusion.

### 3.2. Rituximab Infusion Protocol and Premedication 

Of the 183 treatment episodes collected, 121 were standard protocols, while 62 were split protocols. All patients received premedication consisting of an antihistamine, an antipyretic and a glucocorticoid, with only slight variations. All patients received acetaminophen as an antipyretic and the administered dose was 650 mg in over 96% of the cases. Glucocorticoid type, dosage, and route of administration varied according to chemotherapy protocols and the healthcare center’s practices. Only one patient received ranitidine as an antihistamine, while others received 25 or 50 mg of diphenhydramine, orally or intravenously.

### 3.3. Symptoms, Clinical Signs, and Management of Infusion-Related Reactions

The most common signs and symptoms associated with IRR were flushing (38%), nausea and/or vomiting (29%), hypotension (28%), chills (27%), and hypertension (23%) (Table 2). No patients died from an IRR. During an IRR, the most common intervention was to stop the infusion and resume at a slower rate once the symptoms improved (36%). In 27% of the cases, the infusion was continued despite the IRR. Antihistamine diphenhydramine (40%), glucocorticoid (31%), and oxygen (20%) were the most common supportive care measures administered during an IRR.

### 3.4. Incidence and Severity of Infusion-Related Reactions

**Figure 1:**
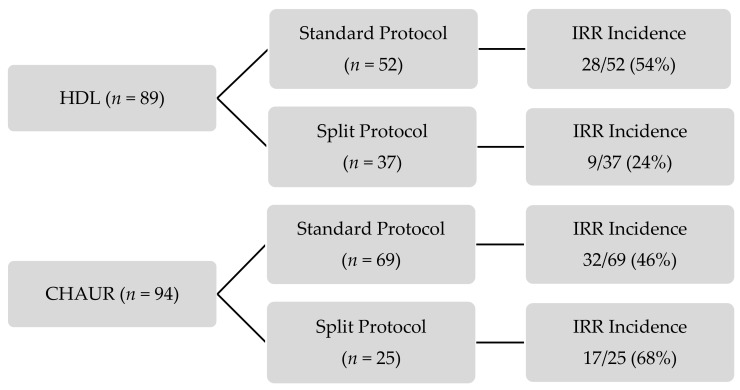
Incidence of infusion-related reactions in each group. CHAUR: Centre hospitalier affilié universitaire régional; HDL: Hôtel-Dieu de Lévis; IRR: infusion-related reactions.

The incidence of IRR in each group is presented in Figure 1. All IRR occurred on day 1 of fractionated dosing protocols. More than half of the patients did not experience an IRR. For the standard dosing schedule, the incidence of IRR was similar in both centers; IRR occurred in 54% of HDL’s patients and 46% of CHAUR’s patients.

The results of the primary outcome are presented in Table 3. IRR incidence did not differ significantly between the patients receiving a fractionated dosing schedule and those receiving the standard protocol (42% vs. 50%, *p* = 0.540). However, IRR were significantly less frequent (reduction of 68% in the relative risk) in patients receiving the HDL fractionated dosing schedule compared with CHAUR (24% vs. 68%, *p* = 0.003) (Table 4). The majority of IRR were mild to moderate, with only 15 out of 86 (17%) being severe. No statistically significant difference was observed in IRR severity between either groups (Table 5).

### 3.5. Sensitivity Analysis

We conducted three sensitivity analyses for each outcome to assess the possible effects of prior rituximab exposure, a higher WBC threshold (≥ 50.0 × 10^9^/L), and the presence of comorbid autoimmune hemolytic anemia. The findings were generally consistent with those of the standard analysis except in rituximab-naïve patients, as no statistically significant difference was detected between fractionated dosing schedules in either centers. In these patients, IRR incidence was 33% with the HDL schedule compared with 63% for CHAUR (adjusted RR, 0.41; 95% CI: 0.10–1.78, *p* = 0.236).

### 3.6. Exploratory Results 

#### Infusion-Related Reaction Risk Factors

When standard protocol groups were compared to fractionated dosing schedule groups, we observed that patients with a reduced estimated glomerular filtration rate adjusted for body surface area seemed to be at higher risk of developing an IRR (*p* = 0.0623). In contrast, prior rituximab exposure seemed to be protective (*p* = 0.0166). When both fractionated dosing groups were compared, lower hemoglobin level (*p* = 0.0129), lower dexamethasone equivalent dose in premedication (*p* = 0.0496), and higher WBC count (*p* = 0.0366) seemed to be associated with a greater risk of developing an IRR.

## 4. Discussion

To our knowledge, this is the first study assessing the efficacy of split dosing during the first rituximab infusion to reduce the incidence of IRR among patients with a high number of circulating malignant cells. 

**Figure 2:**
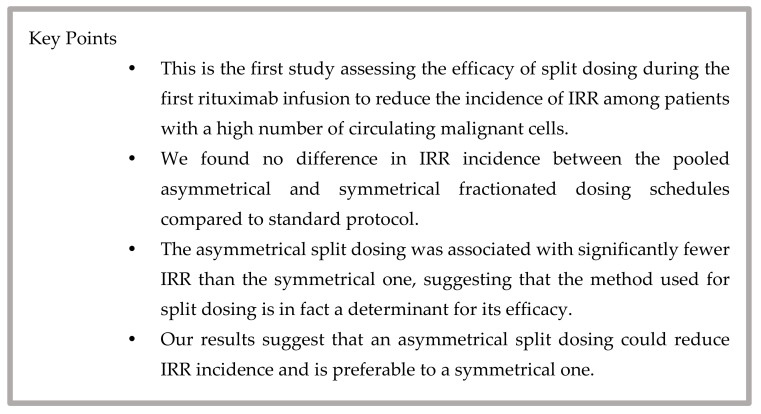
Key Points. IRR: Infusion-related reactions.

In this retrospective observational cohort study, we found that fractionated dosing schedules of both healthcare centers combined did not reduce IRR incidence compared to the standard protocol. Additionally, IRR severity from both fractionated dosing schedules combined did not differ significantly from the standard protocol. However, the fractionated dosing schedule of HDL was associated with significantly fewer IRR than CHAUR’s (24% vs. 68%, *p* = 0.003). The absence of difference between split and standard dosing suggests that CHAUR’s symmetrical split dosing attenuated the results of HDL’s asymmetrical dosing schedule, indicating that not all split dosing protocols are equivalent and that the method used for split dosing is, in fact, a determinant for its efficacy. Thus, an asymmetrical split dosing like the one used in HDL seems preferable to a symmetrical split dosing to prevent IRR. The key points of this study are presented in Figure 2. 

No specific fractionated dosing schedule is currently recommended in the literature, but asymmetrical dosing is the most discussed. For instance, Winkler et al. employed an asymmetrical split dosing over 3 days as follows: 50 mg/m^2^ on day 1, 150 mg/m^2^ on day 2, and 175 mg/m^2^ on day 3 [11]. Despite this fractionated dosing schedule, they found that patients with WBC ≥ 50.0 × 10^9^/L experienced much more severe IRR than other patients. Byrd et al. suggested giving a small dose of rituximab on day 1 (100 mg) and the remaining dose of the 375 mg/m^2^ on the following day [12]. Fractionated dosing schedules have also been studied with other monoclonal antibodies used in hematologic cancers and have caused IRR. Rifkin et al. assessed the efficacy and safety of a symmetrical fractionated dosing schedule (8 mg/kg on days 1 and 2) of daratumumab, an antibody directed against the CD38 antigen with similar precautions as rituximab, to prevent IRR. No statistical difference was observed between the incidence of IRR in the split first dose group and the single-dose group (16 mg/kg on day 1) (47.8% vs. 48.3%) [15]. Obinutuzumab is another anti-CD20 antibody, for which an asymmetrical split dosing is recommended for the first infusion, i.e., 100 mg on day 1 and 900 mg on day 2 [16].

The most frequent risk factor for developing an IRR reported in the literature is a high lymphocyte count [1,9,11,12]. In this study, the mean WBC was significantly higher in the fractionated dosing schedule groups than standard protocol groups (114.6 × 10^9^/L vs. 72.4 × 10^9^/L) and was significantly higher in patients receiving CHAUR’s fractionated dosing schedule compared to HDL (198.7 × 10^9^/L vs. 71.3 × 10^9^/L). The propensity score used to adjust our results helped limit the impact of WBC imbalance between the groups. When comparing IRR incidence between fractionated dosing schedules in each center, the adjusted results accounting for this imbalance still favored HDL’s protocol (see Table 4). In the sensitivity analysis exploring a different WBC threshold (≥50.0 × 10^9^/L), we did not find a significant difference in IRR incidence compared to the WBC threshold of ≥25.0 × 10^9^/L. The exact WBC threshold at which the risk of developing an IRR significantly increases remains unknown. 

A slow and gradual titration infusion rate is another strategy recommended in the literature to reduce rituximab-induced IRR incidence in patients with a high number of circulating malignant cells [7]. The slower titration rate on day 1 of HDL’s fractionated dosing schedule compared to all other protocols in this study might have helped reduce IRR in this group.

Sensitivity analysis performed to assess the possible effects of prior rituximab exposure showed no statistically significant difference in IRR incidence between fractionated dosing schedule groups in either center. This result might be explained by a lack of statistical power, hence the wide confidence intervals. Only 40 patients were included in this analysis. 

One of the strengths of this study was the use of a propensity score. The statistical method allowed adjusting the results according to unbalanced baseline variables between groups, thus, controlling the potential confounding factors. Moreover, to our knowledge, this study has the largest sample size with the aim of assessing the efficacy of rituximab split dosing [11,12]. This study has very few exclusion criteria, which allows the generalization of results and enhances the external validity. Sensitivity analysis also allowed evaluating the impact of different factors on the results. 

This study also has several limitations. Data collection was carried out retrospectively from electronic patient records and was, therefore, dependent on the information available which could be subjective and was written by healthcare professionals. Some information, like signs or symptoms occurring during rituximab administration, or actions taken to alleviate them, could have been missing or incomplete. However, since every rituximab administration has been systematically monitored over the years using a specific monitoring protocol, and since IRR is a dichotomous variable (presence or absence), the risk of information bias was minimal. The risk of information bias was more significant for assessing IRR severity, a secondary outcome since incomplete documentation of medical interventions or missing signs or symptoms could have led to an under or overestimated classification. Data were also collected by two different investigators, one in each healthcare center. Variability in data collection regarding subjective information could have also induced information bias. However, investigators systematically consulted each other when they were in doubt. The use of the NCI-CTCAE scale to determine IRR severity also helped limit bias considering this rating scale is validated and used in multiple studies collecting adverse drug events. This also ensures reproducible results. Additionally, as previously mentioned, the WBC count was used to assess the high tumor burden in this study instead of lymphocyte count. However, considering that lymphocyte count is intrinsically related to WBC count in B cell malignancies, and that mean WBC counts for all groups were dramatically higher than 25 × 10^9^/L, the use of the WBC count should not have affected our results. Furthermore, since premedication with a glucocorticoid is recommended to prevent rituximab-induced IRR [1], the dexamethasone equivalent dose was included in the propensity score when fractionated dosing groups in each center were compared together since their mean dexamethasone equivalent dose was significantly different. Glucocorticoid premedication varied according to chemotherapy protocol, healthcare center, and patient’s characteristics. Since our study spans almost 12.5 years, glucocorticoid premedication also varied through time, especially in HDL, where protocols in the past few years have favored oral over intravenous administration and lower doses. The variation in dosage, molecule, and route of administration could have influenced IRR incidence. Diphenhydramine was the antihistamine used in the premedication for all patients except one. The dosage and administration route also varied according to the healthcare center and patient’s characteristics, but to a smaller extent than glucocorticoids. Differences in antihistamine type, dosage, and administration route were not taken into account in our results. Thereby, limitations associated with the retrospective design of this study ought to be considered in the interpretation of the results. However, the use of a propensity score helped to mitigate the lack of randomization by adjusting results for unbalanced variables at the baseline. The magnitude of the effect observed between the split protocols of HDL and CHAUR is large enough to state with confidence that the results remain significant despite potential bias.

From a time and cost-saving perspective, for a patient with a standard body surface area of 1.73 m^2^, who does not develop an IRR during the first rituximab administration, HDL’s asymmetrical fractionated dosing schedule takes approximately 1.25 h longer than CHAUR’s symmetrical one and a little more than 2 h longer than the standard protocol recommended for a first rituximab infusion. Although HDL’s split protocol takes more time to administer, it might be the most advantageous option considering that it reduced IRR by more than 50% compared to CHAUR’s split protocol. Furthermore, IRR management is time-consuming and requires many resources, and IRR have multiple consequences for patients such as anxiety and fear.

Our results demonstrate that the asymmetrical fractionated dosing schedule reduced IRR risk by 68% compared to the symmetrical one. These findings could change healthcare center practices for those who use a symmetrical split dosing or no split dosing at all for the first rituximab infusion in patients with a high number of circulating malignant cells.

## 5. Conclusions

In conclusion, both fractionated dosing schedules combined did not reduce IRR incidence or the severity of the reactions more effectively than the standard protocol. However, HDL’s fractionated dosing schedule was associated with significantly fewer IRR than CHAUR’s, suggesting that an asymmetrical split dosing could effectively reduce IRR incidence and be preferable to a symmetrical one. More studies, with a larger sample size, will be necessary to clearly demonstrate the efficacy of a split dosing regimen to reduce rituximab-induced IRR and determine the optimal dosing schedule. Future research could complement this study by evaluating the impact of split dosing on subsequent rituximab cycles for patients with high lymphocyte counts.

## Figures and Tables

**Table 1:** Baseline patient characteristics.

Patient Characteristics	HDL (*n* = 89)		CHAUR (*n* = 94)	
	Standard Protocol (*n* = 52)	Fractionated Dosing Schedule (*n* = 37)	Standard Protocol (*n* = 69)	Fractionated Dosing Schedule (*n* = 25)
Women [*n* (%)]	13 (25)	14 (38)	18 (26)	10 (40)
Median age (years) (min–max)	70 (52–87)	68 (46–89)	66 (41–87)	67 (48–81)
Median BMI (kg/m^2^) (min–max)	26 (16–40)	26 (19–40)	25 (18–38)	26 (19–42)
Diagnosis				
CLL [*n* (%)]	46 (89)	33 (89)	50 (73)	22 (88)
NHL [*n* (%)]	6 (12)	4 (11)	19 (28)	3 (12)
Chemotherapy protocol				
FCR [*n* (%)]	11 (21)	11 (30)	24 (35)	9 (36)
BR [*n* (%)]	0 (0)	9 (24)	2 (3)	9 (36)
R [*n* (%)]	14 (27)	6 (16)	13 (19)	3 (12)
R-CVP [*n* (%)]	12 (23)	2 (5)	23 (33)	3 (12)
Other [*n* (%)]	15 (29)	9 (24)	7 (10)	1 (4)
Mean dexamethasone equivalent dose in premedication (mg) (min–max)	14.5 (7.5–25.0)	12.2 (8.0–25.0)	7.8 (3.8–33.8)	7.5 (3.8–26.3)
Prior exposure to rituximab [*n* (%)]	14 (28)	13 (35)	16 (23)	9 (36)
Mean WBC (×10^9^/L) (min–max)	66.2 (25.1–510.0)	71.3 (25.1–451.7)	82.3 (25.0–312.0)	198.7 (31.9–426.0)
Median Hb (g/L) (min–max)	104 (71–152)	114 (75–162)	110 (51–147)	96 (68–131)
Median eGFR adjusted (mL/min) (min–max)	74 (26–106)	80 (40–124)	77 (22–162)	67 (30–122)
Drug allergy [*n* (%)]	5 (10)	5 (14)	11 (16)	5 (20)
Lung condition [*n* (%)]	5 (10)	7 (19)	6 (9)	5 (20)
Heart condition [*n* (%)]	3 (6)	9 (24)	13 (19)	3 (12)
AIHA [*n* (%)]	4 (8)	2 (5)	8 (12)	2 (8)

AIHA: autoimmune hemolytic anemia; BMI: body mass index; BR: bendamustine and rituximab; CHAUR: Centre hospitalier affilié universitaire régional; CLL: chronic lymphocytic leukemia; eGFR adjusted: estimated glomerular filtration rate adjusted for body surface area; FCR: fludarabine, cyclophosphamide, and rituximab; Hb: hemoglobin; HDL: Hôtel-Dieu de Lévis; Heart condition: atrial fibrillation, chronic heart failure, history of myocardial infarction, atherosclerotic cardiovascular disease, or angina; Lung condition: chronic obstructive pulmonary disease, asthma, or pulmonary fibrosis; NHL: non-Hodgkin lymphoma; R: rituximab; R-CVP: rituximab, cyclophosphamide, vincristine, and prednisone; WBC: white blood cell.

**Table 2:** Infusion-related reaction characteristics.

IRR Characteristics	HDL (*n* = 37)	CHAUR (*n* = 49)
Symptoms and clinical signs (>10%) [*n* (%)] Flushing Nausea and/or vomiting Hypotension Chills Hypertension Desaturation Dyspnea Dizziness Pruritus Fever Throat tightness or irritation Chest pain or tightness	13 (35) 13 (35) 11 (30) 9 (24) 12 (32) 7 (19) 5 (14) 2 (5) 5 (14) 2 (5) 1 (3) 2 (5)	20 (41) 12 (25) 13 (27) 14 (29) 8 (16) 9 (18) 7 (14) 9 (18) 3 (6) 6 (12) 6 (12) 5 (10)
Impact of IRR on the ongoing rituximab infusion [*n* (%)] No impact Infusion rate decreased Infusion stopped and resumed at a slower rate Infusion stopped and resumed at the same rate Infusion stopped and not resumed Infusion stopped and postponed Other	11 (30) 0 (0) 9 (24) 10 (27) 4 (11) 3 (8) 0 (0)	12 (25) 2 (4) 22 (45) 9 (18) 3 (6) 0 (0) 1 (2)
Medication given during IRR [*n* (%)] Antipyretic Antihistamine H1 Antihistamine H2 Corticosteroid Bronchodilator Epinephrine Intravenous hydration Oxygen Other	4 (11) 20 (54) 1 (3) 11 (30) 2 (5) 0 (0) 2 (5) 7 (19) 9 (24)	10 (20) 14 (29) 3 (6) 16 (33) 3 (6) 0 (0) 4 (8) 10 (20) 8 (16)

IRR: Infusion-related reactions.

**Table 3:** Incidence of infusion-related reactions according to infusion protocol.

Incidence of IRR	Fractionated Dosing Schedule (HDL + CHAUR) (*n* = 62)	Standard Dosing Schedule (HDL + CHAUR) (*n* = 121)	Relative Risk (95% CI)	Adjusted Relative Risk (95% CI)	Adjusted *p* Value
IRR [*n* (%)]	26 (42%)	60 (50%)	0.84 (0.60–1.19)	0.89 (0.60–1.30)	0.540

CHAUR: Centre hospitalier affilié universitaire régional; CI: confidence interval; HDL: Hôtel-Dieu de Lévis; IRR: infusion-related reactions. Relative risk and *p* value adjusted with the propensity score considering the following unbalanced variables: sex, chemotherapy protocol, comorbid lung condition, white blood cell count.

**Table 4:** Incidence of infusion-related reactions according to the fractionated dosing schedule.

Incidence of IRR	HDL Fractionated Dosing Schedule (*n* = 37)	CHAUR Fractionated Dosing Schedule (*n* = 25)	Relative Risk (95% CI)	Adjusted Relative Risk (95% CI)	Adjusted *p* Value
IRR [*n* (%)]	9 (24%)	17 (68%)	0.36 (0.19–0.67)	0.32 (0.15–0.67)	0.003

CHAUR: Centre hospitalier affilié universitaire régional; CI: confidence interval; HDL: Hôtel-Dieu de Lévis; IRR: infusion-related reactions. Relative risk and *p* value adjusted with the propensity score considering the following unbalanced variables: hemoglobin, mean dexamethasone equivalent dose, white blood cell count.

**Table 5:** Severity of infusion-related reactions according to infusion protocol.

Severity of IRR	Fractionated Dosing Schedule (*n* = 26)	Standard Dosing Schedule (*n* = 60)	Relative Risk (95% CI)	Adjusted Relative Risk (95% CI)	Adjusted *p* Value
Severity of IRR					
Mild to moderate [*n* (%)]	20 (77%)	51 (85%)			
Severe [*n* (%)]	6 (23%)	9 (15%)	1.54 (0.61–3.88)	0.94 (0.27–3.26)	0.920

CI: confidence interval; IRR: infusion-related reactions. Relative risk and *p* value adjusted with the propensity score considering the following unbalanced variables: sex, chemotherapy protocol, comorbid lung condition, white blood cell count.

## Data Availability

The data are not publicly available to protect the privacy of the patients. The data are available upon request from the corresponding author.
